# Induction of ATF4-Regulated Atrogenes Is Uncoupled from Muscle Atrophy during Disuse in Halofuginone-Treated Mice and in Hibernating Brown Bears

**DOI:** 10.3390/ijms24010621

**Published:** 2022-12-30

**Authors:** Laura Cussonneau, Cécile Coudy-Gandilhon, Christiane Deval, Ghita Chaouki, Mehdi Djelloul-Mazouz, Yoann Delorme, Julien Hermet, Guillemette Gauquelin-Koch, Cécile Polge, Daniel Taillandier, Julien Averous, Alain Bruhat, Céline Jousse, Isabelle Papet, Fabrice Bertile, Etienne Lefai, Pierre Fafournoux, Anne-Catherine Maurin, Lydie Combaret

**Affiliations:** 1 Université Clermont Auvergne, INRAE, UNH UMR 1019, CRNH Auvergne, 63000 Clermont-Ferrand, France; 2 Centre National d’Etudes Spatiales, CNES, 75001 Paris, France; 3 Université de Strasbourg, CNRS, IPHC UMR 7178, 67037 Strasbourg, France

**Keywords:** skeletal muscle, unloading, hindlimb suspension, halofuginone, ATF4, TGF-β/BMP signalling, hibernating bear, atrogenes, muscle atrophy

## Abstract

Activating transcription factor 4 (ATF4) is involved in muscle atrophy through the overexpression of some atrogenes. However, it also controls the transcription of genes involved in muscle homeostasis maintenance. Here, we explored the effect of ATF4 activation by the pharmacological molecule halofuginone during hindlimb suspension (HS)-induced muscle atrophy. Firstly, we reported that periodic activation of ATF4-regulated atrogenes (*Gadd45a*, *Cdkn1a*, and *Eif4ebp1*) by halofuginone was not associated with muscle atrophy in healthy mice. Secondly, halofuginone-treated mice even showed reduced atrophy during HS, although the induction of the ATF4 pathway was identical to that in untreated HS mice. We further showed that halofuginone inhibited transforming growth factor-β (TGF-β) signalling, while promoting bone morphogenetic protein (BMP) signalling in healthy mice and slightly preserved protein synthesis during HS. Finally, ATF4-regulated atrogenes were also induced in the atrophy-resistant muscles of hibernating brown bears, in which we previously also reported concurrent TGF-β inhibition and BMP activation. Overall, we show that ATF4-induced atrogenes can be uncoupled from muscle atrophy. In addition, our data also indicate that halofuginone can control the TGF-β/BMP balance towards muscle mass maintenance. Whether halofuginone-induced BMP signalling can counteract the effect of ATF4-induced atrogenes needs to be further investigated and may open a new avenue to fight muscle atrophy. Finally, our study opens the way for further studies to identify well-tolerated chemical compounds in humans that are able to fine-tune the TGF-β/BMP balance and could be used to preserve muscle mass during catabolic situations.

## 1. Introduction

Many unloading conditions (e.g., microgravity, bed rest, or physical inactivity) lead to a loss of muscle mass and strength. This muscle atrophy is associated with adverse health effects such as autonomy decline and increased morbidity and mortality [1,2,3]. Considering the lack of proven, easy-to-use therapeutic or preventive treatment, muscle atrophy remains a major public health issue (World Health Organisation data) [4].

The underlying molecular mechanisms of muscle atrophy involve the dysregulation of a complex network of intracellular pathways leading to an imbalance in protein turnover [5,6,7,8,9]. Activating transcription factor 4 (ATF4) is overexpressed in many conditions of muscle atrophy [10,11,12,13,14] and is, therefore, considered as an atrogene, i.e., one of the genes with expression at the mRNA level that is commonly altered during atrophy [14]. ATF4 belongs to the integrated stress response (ISR) pathway, a conserved intracellular network activated in response to various intrinsic and extrinsic stresses (e.g., amino acid (AA) depletion and endoplasmic reticulum (ER) stress) to restore cellular homeostasis [15]. Activation of the ISR involves phosphorylation of eukaryotic translation initiation factor 2 (eIF2α) by several kinases (i.e., general control nonderepressible 2 (GCN2), protein kinase RNA-like ER kinase (PERK), protein kinase R (PKR), heme-regulated inhibitor (HRI), and microtubule affinity-regulating kinase 2 (MARK2)), resulting in the global inhibition of protein synthesis but the increased translation of certain mRNAs, including ATF4 [15,16]. Inhibition of ATF4 in skeletal muscle limits starvation-, immobilisation-, and ageing-induced atrophy, whereas ATF4 induction results in muscle wasting [11,12,13,17]. ATF4 target genes include some atrogenes, such as GADD45A and CDKN1A, that are required for ATF4-mediated muscle atrophy [11,12,13,18], and TRIB3, which is involved in fasting- and ageing-induced muscle atrophy [19,20]. However, ATF4 targets also include genes that may be involved in the maintenance of muscle homeostasis. Indeed, ATF4 contributes to the transcription of autophagy-related genes [21,22,23,24] and is activated during mitochondrial perturbations (e.g., oxidative stress) to restore mitochondrial homeostasis [25,26]. In fact, activation of the eIF2α-ATF4 pathway by the pharmacological molecule halofuginone (HF), prior to stressful events (i.e., ischemia-reperfusion injury models) has shown positive effects on the preservation of kidney and liver function [27]. Moreover, the maintenance of autophagy and mitochondria homeostasis are essential for maintaining muscle mass [28,29,30]. Altogether, this led to the hypothesis that the ATF4 pathway may have a dual role in skeletal muscle. 

Interestingly, halofuginone also improved muscle performance during dystrophies, mainly through its antifibrotic properties [31,32,33,34]. Whether these beneficial effects involve a regulation of the ATF4 pathway has never been investigated. However, they mainly involved the inhibition of the transforming growth factor-β (TGF-β) pathway [35,36,37]. The TGF-β signalling pathway acts as a negative regulator of muscle mass, notably through the transcriptional induction of the atrogenes TRIM63/MuRF1 and FBXO32/Atrogin-1 [38,39,40]. When inhibited, it promotes a profound muscle hypertrophy phenotype in various conditions and species [41,42]. Members of TGF-β signalling belong to the TGF-β superfamily [38,39], as do the bone morphogenetic protein (BMP) signalling members. The BMP signalling pathway [39,43] instead acts as a positive regulator of muscle mass through the transcriptional repression of the atrogene FBXO30/Musa1, which is required for denervation-induced muscle loss [44,45]. When inhibited, it profoundly exacerbates denervation-induced muscle atrophy [44,45].

This study aimed to explore the impacts of HF-induced ATF4 signalling on skeletal muscle under basal conditions and hindlimb suspension-induced atrophy in mice. We further deciphered the molecular mechanisms of HF by focusing on protein metabolism and TGF-β/BMP signalling. We also took advantage of a model of muscle atrophy resistance that we previously explored [46], and studied the regulation of the ATF4-induced atrogenes in hibernating brown bear muscle. 

## 2. Results

### 2.1. Induction of ATF4-Regulated Atrogenes Does Not Affect Muscle Mass in Mice

**Figure 1:**
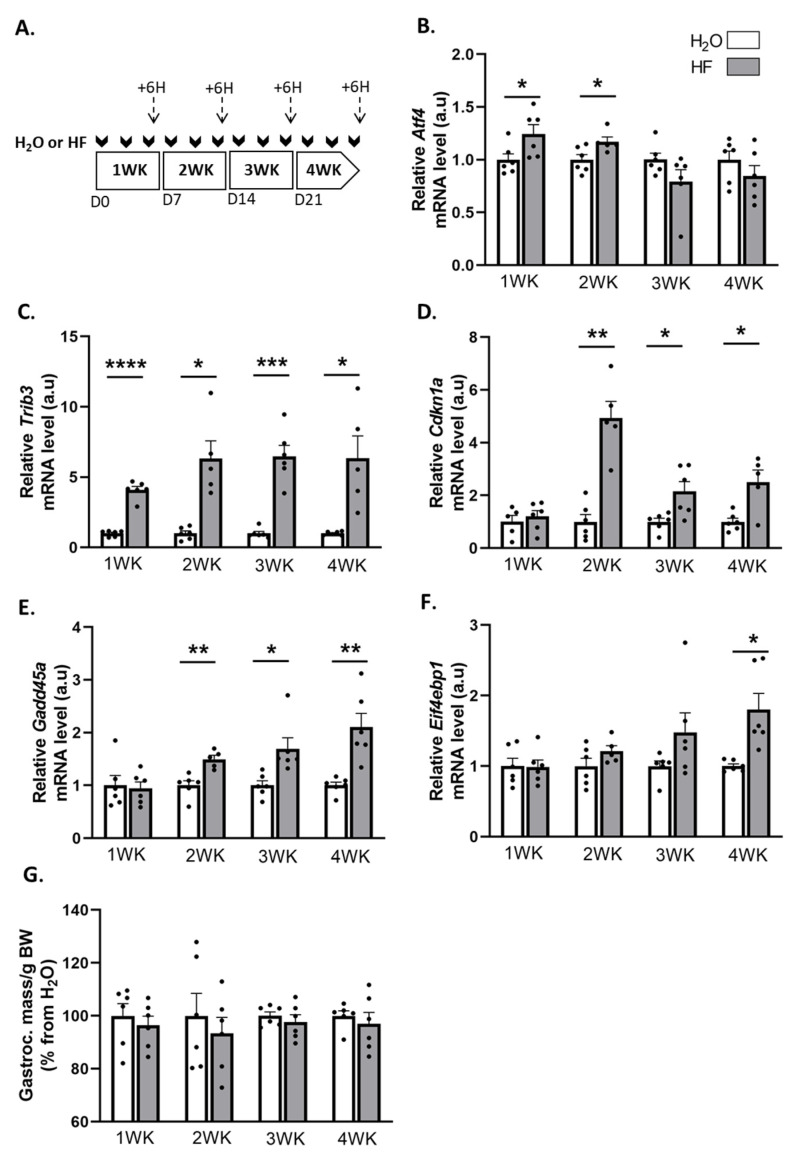
Halofuginone activates the expression of ATF4-regulated atrogenes in muscle without leading to atrophy. (**A**) Schematic representation of the experimental protocol, where mice received H_2_O (white bars) or HF (0.25 µg/g, grey bars) 3 times a week for up to 4 weeks (WK). Muscles were collected 6 h after the last HF administration at the end of each week (dotted arrows). (**B**–**F**) Relative mRNA levels in gastrocnemius for *Atf4*, *Trib3*, *Cdkn1a*, *Gadd45a*, and *Eif4ebp1* were measured by RT-qPCR. Data were normalised using *Tbp*. Data are expressed as fold change vs. H_2_O within each week and are presented as individual values with mean bars ± SEM. (**G**) Gastrocnemius muscle mass per gram of body weight (BW). Data are expressed as a percentage from H_2_O within each week and presented as individual values with mean bars ± SEM. Statistics are described in Section 4. * *p*_adj_ < 0.05; ** *p*_adj_ < 0.01; *** *p*_adj_ < 0.001; **** *p*_adj_ < 0.0001.

We used halofuginone (HF) to induce ATF4 transcriptional activity in mouse muscles, and we investigated the effect on skeletal muscle mass. For that purpose, mice were treated with HF three times a week for up to 4 weeks. Six hours after the last HF administration at the end of each week, we measured the mRNA levels for some ATF4 target genes, involved in muscle atrophy, i.e., *Trib3*, *Cdkn1a*, *Gadd45a*, and *Eif4ebp1* (Figure 1A–F). Except for *Atf4*, for which mRNA levels were elevated during the first 2 weeks of HF treatment, *Trib3*, *Cdkn1a*, and *Gadd45a* were all overexpressed in muscles after 2 weeks of HF treatment compared to H_2_O-treated mice. Of note, *Eif4ebp1* mRNA levels increased in mouse muscles after 4 weeks of HF-treatment compared to H_2_O, with a noticeable trend after 3 weeks of treatment (Figure 1F). We also investigated the regulation of other ATF4 target genes and showed the overexpression of *Asns* over the 4 weeks of HF treatment as well as a trend for *Ddit3* and *Ppp1r15a* (Supplementary Figure S1). 

These data underline an activation of the ATF4 transcriptional activity. However, despite the overexpression of the ATF4-regulated atrogenes, the mass of the gastrocnemius, soleus, tibialis anterior, and extensor digitorum longus (EDL) muscles remained unchanged during the 4 weeks of HF treatment (Figure 1G, Supplementary Figure S1). Altogether, these data suggest that a long-term halofuginone administration induced ATF4-regulated atrogenes without leading to muscle atrophy. 

### 2.2. Overexpression of ATF4-Regulated Atrogenes during Hindlimb Suspension Is Uncoupled from Muscle Atrophy in HF-Treated Mice

**Figure 2:**
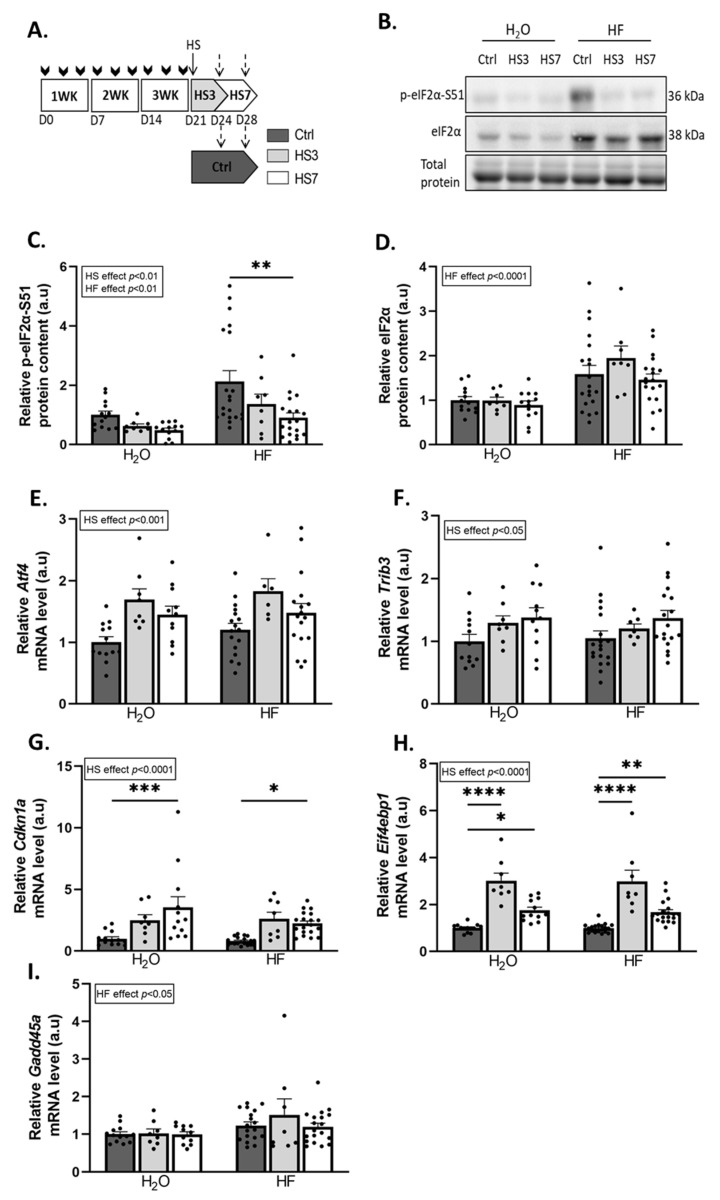
Hindlimb suspension induces ATF4 pathway. (**A**) Schematic representation of the experimental protocol, where mice received H_2_O or halofuginone (HF) oral administration (0.25 µg/g) 3 times a week for 3 weeks (WK) (black arrows) and were then subjected to hindlimb suspension for 3 (HS3, light grey bars) or 7 (HS7, white bars) days or kept unsuspended (Ctrl, dark grey). The dotted arrows represent the time when the muscles were collected. (**B**–**D**) Relative protein levels in gastrocnemius for phosphorylated and total eIF2α were measured by Western blotting, quantified, and normalised to the total protein content. Representative Western blots are shown. (**E**–**I**) Relative mRNA levels in gastrocnemius for *Atf4*, *Trib3*, *Cdkn1a, Eif4ebp1*, and *Gadd45a* were measured by RT-qPCR and were normalised using *Tbp.* Data are expressed as fold change vs. H_2_O-Ctrl and are presented as individual values normalised mean bars ± SEM. Statistics are described in Section 4. * *p*_adj_ < 0.05; ** *p*_adj_ < 0.01; *** *p*_adj_ < 0.001; **** *p*_adj_ < 0.0001.

We then investigated the effect of the induction of the ATF4 pathway by HF treatment during the muscle atrophy induced by hindlimb suspension (HS). Briefly, mice received HF three times a week for 3 weeks and were then, 3 days after the last HF administration, hindlimb-suspended or not for 3 or 7 days (Figure 2A). Measurements were thus performed at least 6 days after the last HF administration. HF induces ATF4 activation through the phosphorylation of eIF2α [47]. We observed that HF treatment resulted in overall higher phosphorylated and total eIF2α protein levels compared to H_2_O-treated mice (Figure 2B–D). In addition, HS led to an overall decrease in phosphorylated eIF2α protein levels compared to the controls (Ctrls) (Figure 2B,C). In addition, levels of the mRNA encoding the phosphatase GADD34 (*Ppp1r15a*) were higher at 3 days of hindlimb suspension (HS3) compared to the Ctrls in both H_2_O- and HF-treated mice (Supplementary Figure S2). The expression of ATF4-regulated genes was not different between Ctrl-HF and Ctrl-H_2_O groups (Figure 2 and Supplementary Figure S2). The mRNA levels of *Atf4* and its target genes involved in muscle atrophy, i.e., *Trib3*, *Cdkn1a*, and *Eif4ebp1,* were higher during HS in both H_2_O- and HF-treated mice (Figure 2E–H), while the mRNA levels of the ATF4-regulated atrogene *Gadd45a* remained unchanged (Figure 2I). Of note, mRNA expression of other ATF4 target genes remained unchanged for *Asns* or slightly decreased upon HS for *Ddit3*. (Supplementary Figure S2). Altogether, Figure 1 and Figure 2 shows that (i) HF administration induced ATF4-regulated atrogenes after 6 h, but no more after 6 days, indicating that this effect was rapid and transient, and (ii) HS induced overexpression of these atrogenes.

**Figure 3:**
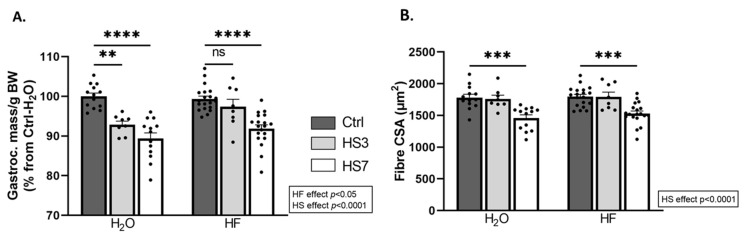
Halofuginone treatment prior to hindlimb suspension mitigates atrophy in gastrocnemius muscle. Mice were treated with H_2_O or halofuginone (HF, 0.25 µg/g) 3 times a week for 3 weeks and were then subjected to hindlimb suspension for 3 (HS3, light grey bars) or 7 (HS7, white bars) days or kept unsuspended (Ctrl, dark grey bars), as described in Figure 2A. (**A**) Gastrocnemius muscle mass per gram of body weight (BW). Data are expressed as a percentage from H_2_O-Ctrl and presented as individual values with mean bars ± SEM. (**B**) Mean fibre cross-sectional area in gastrocnemius muscle. Data are presented as individual values with mean bars ± SEM. Statistics are described in Section 4. ** *p*_adj_ < 0.01; *** *p*_adj_ < 0.001; **** *p*_adj_ < 0.0001; ns = non-significant.

We next investigated the outcomes on skeletal muscle. Gastrocnemius muscle had atrophied only in H_2_O-treated mice after 3 days of HS (H_2_O-HS3) and in both H_2_O- and HF-treated mice after 7 days of HS. Surprisingly, the average of the fibre cross-sectional area (CSA) did not change in H_2_O-HS3 mice compared to the Ctrls but was lower after HS7 regardless of the treatment (Figure 3B). We further analysed the distribution of gastrocnemius fibre CSA (Supplementary Figure S3A,B). We reported (i) a lower proportion of small fibres and (ii) a higher proportion of large fibres in HF-treated mice compared to the H_2_O group (Supplementary Figure S3A). This observation was restricted to fast-twitch fibres (i.e., 2X/2B) (Supplementary Figure S3B). Taken together, our data suggest that induction of ATF4-regulated atrogenes is not associated with muscle atrophy after 3 days of hindlimb suspension in HF-treated mice and, thus, that HF slightly preserves muscle mass during HS.

### 2.3. Halofuginone Treatment Inhibits TGF-β While Promoting BMP Signalling in Gastrocnemius Muscle

**Figure 4:**
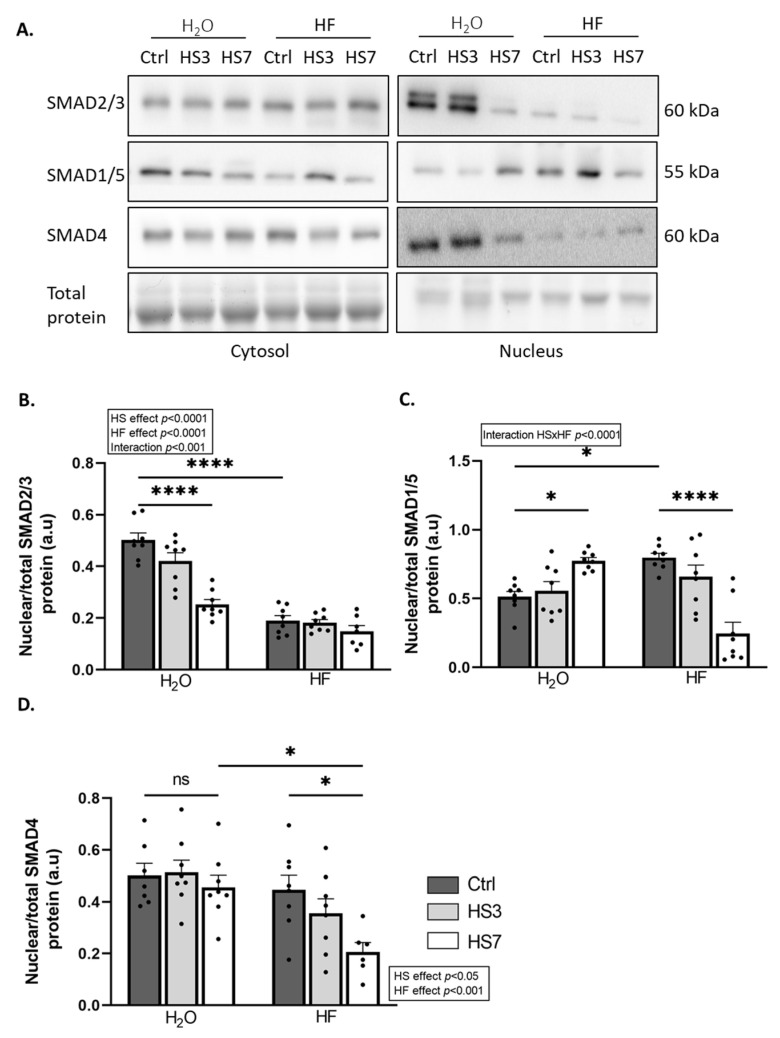
Halofuginone treatment inhibits TGF-β while promoting BMP signalling in gastrocnemius muscle. Mice were treated with H_2_O or halofuginone (HF, 0.25 µg/g) 3 times a week for 3 weeks and were then subjected to hindlimb suspension for 3 (HS3, light grey bars) or 7 (HS7, white bars) days or kept unsuspended (Ctrl, dark grey bars), as described in Figure 2A. (**A**–**D**) The ratio of protein levels in gastrocnemius for the transcription factors SMAD2/3 (TGF-β signalling), SMAD1/5 (BMP signalling), and SMAD4 (TGF-β and BMP signalling) have been assessed in the nuclear and cytosolic subcellular fractions, quantified, and normalised to the total protein content. Representative Western blots are shown. The ratio of nuclear SMAD contents on the total (cytosolic and nuclear) SMAD content was calculated. Data are expressed as fold change vs. H_2_O-Ctrl and presented as individual values with mean bars ± SEM. Statistics are described in Section 4. * *p*_adj_ < 0.05; **** *p*_adj_ < 0.0001.

HF inhibits the TGF-β pathway [36,48]. We and others have recently reported that inhibition of the TGF-β signalling is associated with the concomitant activation of BMP signalling [45,46]. Therefore, we investigated how HF treatment and subsequent HS treatment affected these pathways in skeletal muscle. The nuclear localisation of SMADs mirrors the upstream activation of the TGF-β or BMP pathway [49]. We, thus, measured protein levels for the transcription factors SMAD2/3 (TGF-β signalling), SMAD1/5 (BMP signalling), and SMAD4 (TGF-β and BMP signalling) in nuclear and cytosolic fractions (Figure 4A–D and Supplementary Figure S4A–F). The ratio of nuclear SMAD2/3 to total SMAD2/3 was very low in HF-treated mice compared to H_2_O-treated mice and decreased upon HS only in H_2_O-treated mice (Figure 4A,B). Consistently, the overall mRNA levels of several collagens, which are well-known target genes of TGF-β signalling, decreased upon HS (Supplementary Figure S5). Moreover, the ratio of nuclear SMAD1/5 to total SMAD1/5 was higher in HF-Ctrl mice than in H_2_O-Ctrl mice. This ratio was reduced at HS7 compared to the Ctrl in HF-treated mice, while it was increased in H_2_O-treated mice (Figure 4A,C). Finally, the ratio of nuclear SMAD4 to total SMAD4 was overall lower in HF- vs. H_2_O-treated mice (Figure 4A–D), with a decrease in HF-treated mice at HS7 compared to the Ctrl. 

**Figure 5:**
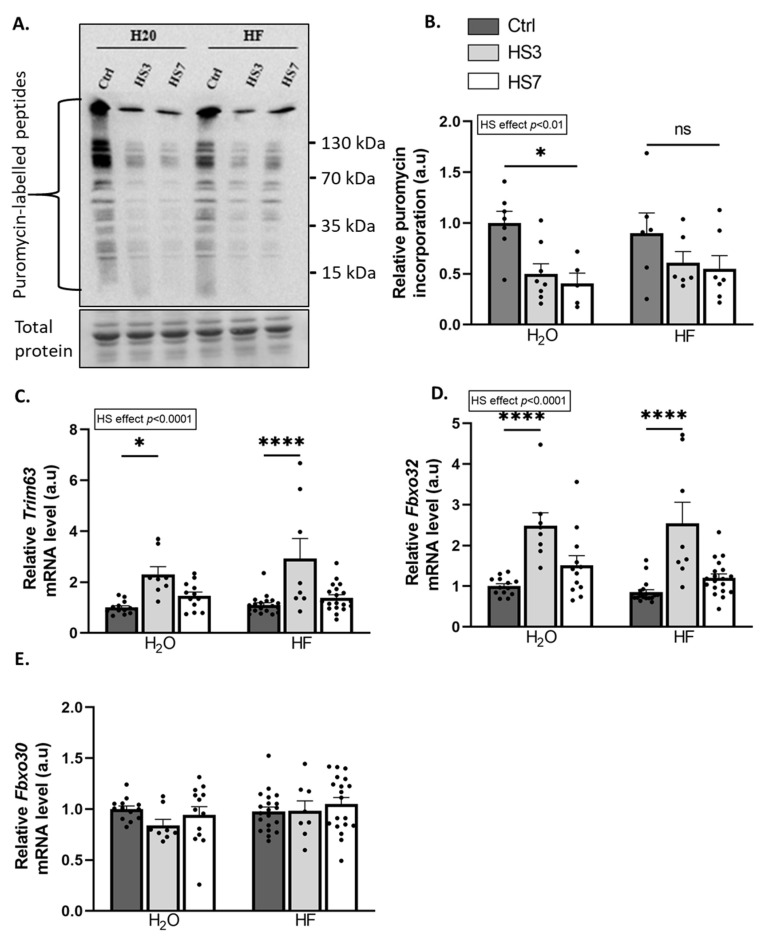
Halofuginone treatment prior to hindlimb suspension partially prevents the decrease in protein synthesis in gastrocnemius muscle. Mice were treated with H_2_O or halofuginone (HF, 0.25 µg/g) 3 times a week for 3 weeks and were then subjected to hindlimb suspension for 3 (HS3, light grey bars) or 7 (HS7, white bars) days or kept unsuspended (Ctrl, dark grey bars), as described in Figure 2A. (**A**,**B**) Relative puromycin incorporation into gastrocnemius muscle was assessed by Western blotting, quantified, and normalised to the total protein content. A representative Western blot is shown. (**C**–**E**) Relative mRNA levels in gastrocnemius for *Trim63*, *Fbxo32*, and *Fbxo30* were measured by RT-qPCR. Data were normalised using *Tbp*. Data are expressed as fold change vs. H_2_O-Ctrl and presented as individual values with mean bars ± SEM. Statistics are described in Section 4. * *p*_adj_ < 0.05; **** *p*_adj_ < 0.0001, or ns = non-significant.

TGF-β catabolic action involves the inhibition of protein synthesis [40,50,51], whereas the anabolic action of BMP involves its promotion [44]. The overall protein-synthesis rates measured by puromycin incorporation were reduced during hindlimb suspension. However, this decrease was only significant in H_2_O-HS7 compared to H_2_O-Ctrl mice (Figure 5A,B). TGF-β signalling acts also as a negative regulator of muscle mass through the induction of the atrogenes TRIM63/MurF1 and FBXO32/Atrogin-1 [38,39], while BMP signalling acts as a positive regulator with the transcriptional repression of the atrogene FBXO30/Musa1 [44,45]. *Trim63* and *Fbxo32* mRNA levels were upregulated only at HS3 in both H_2_O and HF-treated mice, while mRNA levels for the atrogene *Fbxo30* remained unchanged (Figure 5C–E). Our data suggest that while HF inhibits TGF-β signalling, it also promotes BMP signalling in the control gastrocnemius muscles. We also showed that HF partially attenuates the drop in protein synthesis during hindlimb suspension.

### 2.4. ATF4-Regulated Atrogenes Are Overexpressed in Atrophy-Resistant Hibernating Brown Bear Muscle

**Figure 6:**
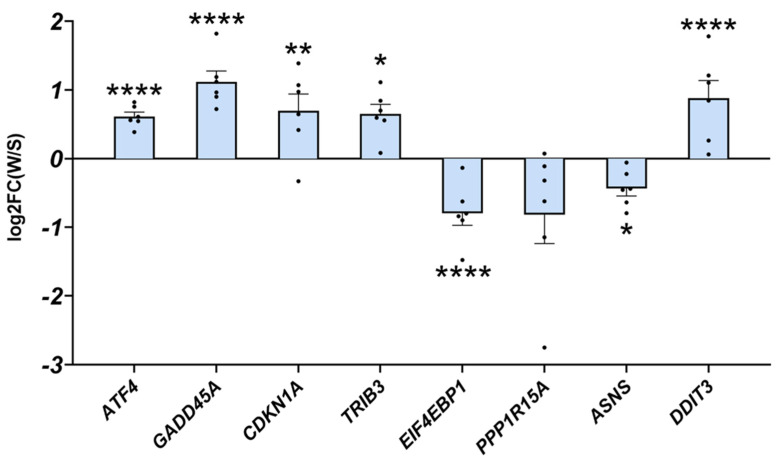
ATF4-regulated atrogenes are induced in atrophy-resistant hibernating brown bear muscles. Gene expression levels for *ATF4*, *GADD45A*, *CDKN1A*, *TRIB3*, *EIF4EBP1*, *PPP1R15A*, *ASNS*, and *DDIT3* in vastus lateralis muscle of active and hibernating brown bears (n = 6 bears/season, the same individuals were sampled and analysed in summer and winter, log2FC winter/summer). Data are presented as individual values as log2FC with mean bars ± lfcSE (log2 fold change standard error). Statistics are described in [46]. * *p*_adj_ < 0.05; ** *p*_adj_ < 0.01; **** *p*_adj_ < 0.0001. FC: fold change; W: winter (hibernating season); S: summer (active season).

Our data strongly suggest that the induction of ATF4 signalling is not always associated with muscle atrophy, either in basal conditions or in HS-induced muscle atrophy. We took advantage of a natural model, i.e., the hibernating brown bear, which experiences only a moderate loss of muscle protein content while remaining completely inactive for up to 6 months [52,53,54]. As with HF treatment, we recently reported a concomitant TGF-β pathway inhibition and BMP pathway activation in hibernating brown bear muscle [46]. We, thus, explored whether ATF4-regulated atrogenes may also be induced in this model. Interestingly, as shown in Figure 6, *Atf4* was upregulated in hibernating brown bear muscle compared to the active counterpart. In addition, the two main ATF4-regulated atrogenes (*Gadd45a* and *Cdkn1a*) and *Trib3* were also induced in hibernating brown bear muscle. Other ATF4 target genes were either down- (*Eif4ebp1*, *Ppp1r15a*, and *Asns*) or upregulated (*Ddit3*). These data show that ATF4-regulated atrogenes are induced in hibernating brown bear muscle, even if they are resistant to atrophy.

## 3. Discussion

**Figure 7:**
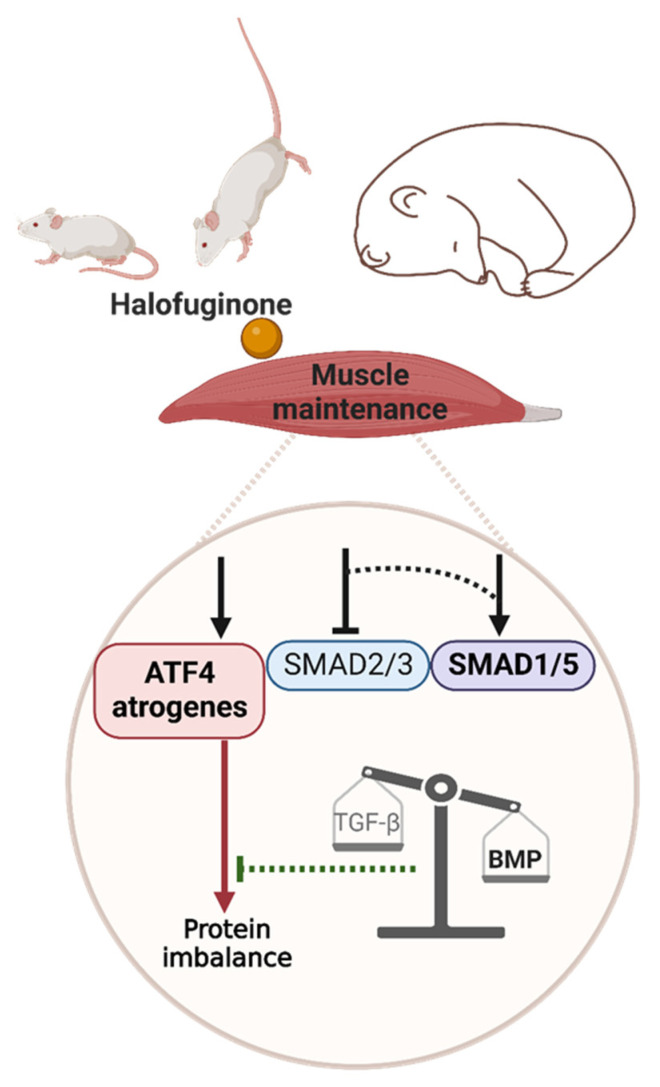
Graphical abstract. The red and green lines represent catabolic and anabolic effects, respectively. Dotted lines represent hypothetical connections. The arrows/T bars above the ATF4 atrogenes, SMAD2/3, and SMAD1/5 boxes represent the induction/inhibition by halofuginone or by an as-yet-unknown mechanism in mouse or bear muscle, respectively. Created with BioRender.com.

Several muscle-wasting conditions, including fasting or physical inactivity, are associated with eIF2α phosphorylation [55,56] and/or ATF4 overexpression, which trigger muscle atrophy [10,11,12,13,18,57]. Moreover, muscle atrophy is hampered during fasting or ageing in mice with reduced ATF4 expression or expressing a phosphorylation-resistant form of eIF2α [11,12,13]. Both CDKN1A and GADD45A are referred to as atrogenes and are required for ATF4-mediated muscle atrophy [11,12,13,18], and TRIB3 is another ATF4 target gene involved in fasting- and ageing-induced muscle atrophy [19,20]. We showed here that overexpression of ATF4-regulated atrogenes was dissociated from muscle wasting (1) in a basal condition, (2) during hindlimb suspension, and (3) in a natural model of muscle-atrophy resistance (Figure 7).

We first observed that the overexpression of the ATF4-regulated atrogenes *Trib3*, *Cdkn1a Gadd45a, and Eif4ebp1*, as well as *Atf4* itself, induced by halofuginone treatment for up to 4 weeks, did not coincide with atrophy in all hindlimb muscles, including gastrocnemius. Subsequently, we then reported that pre-treatment with halofuginone mitigated the atrophy of the gastrocnemius muscle during hindlimb suspension. These positive effects of halofuginone treatment are consistent with reports showing that this dose (i.e., 0.25 µg/g) and frequency of administration (i) were very well-tolerated in mice for up to 3 months and (ii) improved muscle-cell survival, promoted membrane repair, and improved muscle performances in models of muscular dystrophies [31,33,58,59,60]. However, none of these studies explored whether the potential effect of HF would involve the ATF4 pathway. Here, we showed that induction of ATF4-regulated atrogenes was uncoupled from muscle atrophy during hindlimb suspension. Indeed, although ATF4-regulated atrogenes were overexpressed during hindlimb suspension, halofuginone-treated mice displayed a partial preservation of gastrocnemius muscle mass and CSA. In addition, we took advantage of a natural model of resistance to muscle atrophy to examine the expression of ATF4-regulated atrogenes. The brown bear remains completely inactive during hibernation for up to 6 months but, surprisingly, is not sensitive to muscle atrophy [52,53,54], which provides an interesting model for finding new molecular mechanisms to fight muscle atrophy in humans. We showed that the atrogenes *CDKN1A*, *GADD45A*, *ATF4* itself, and *TRIB3* were upregulated in atrophy-resistant hibernating brown bear muscle compared to active bear muscle. Of note, ATF4 is mainly regulated at the translational level [15]. However, in most of the previous studies on the topic, the authors only measured *Atf4* mRNA expression and expression of its target genes as evidence of its activity in skeletal muscle. Indeed, endogenous ATF4 protein cannot be reliably detected in skeletal muscle, presumably due to its low abundance, very short half-life, and lack of a high quality antibody [11,12,13,17,18,61]. Altogether, these data strongly suggest that the induction of the ATF4 pathway can be dissociated from muscle atrophy. ATF4 target genes are highly dependent on the type and duration of stress stimuli [21,62], and the ability to restore homeostasis may be overwhelmed when the stress is too severe or sustained, resulting in cell death through the transcription of pro-apoptotic genes [63,64,65,66]. Therefore, to avoid chronic and acute activation, halofuginone was administrated periodically to activate the eIF2α-ATF4 pathway in mice. In our conditions, the ATF4 transcriptional program may, thus, (i) differ from the transcriptional program induced by a severe and sustained activation and (ii) include genes that might counteract the effect of ATF4-induced atrogenes. 

Halofuginone is well-described to also target the TGF-β pathway [35,36]. The nuclear translocation of the TGF-β transcription factors SMAD2/3 requires the formation of a complex with SMAD4 [43]. Halofuginone-induced eIF2α phosphorylation has been reported to inhibit the nuclear translocation of this complex in intestinal porcine enterocyte cells in vitro [67]. Consistently, we reported here a concomitant overall (i) increase in phosphorylated eIF2α protein levels and (ii) reduction in SMAD2/3 and SMAD4 nuclear protein levels in HF-treated mice. Thus, this highlights in skeletal muscle, for the first time, the possible role of HF-induced eIF2α phosphorylation in TGF-β inhibition. Although, the concomitant collagen downregulation and decrease in SMAD2/3 nuclear protein levels during hindlimb suspension in H_2_O-treated mice suggest a decrease in TGF-β signalling, we cannot exclude that these events are disconnected. Indeed, the SMAD2/3 nuclear protein levels are consistently low in HF-treated mice and, thus, cannot explain the decreased collagen expression during hindlimb suspension. Much remains to be clarified about the mechanisms of action of HF. Indeed, HF is used for its antifibrotic properties mediated by TGF-β inhibition in situations already characterised by fibrosis [48]. This is, however, not the case in our study. In addition, the inhibition of TGF-β signalling in muscles of HF-treated mice could have led to transcriptional changes that remain to be explored. It is possible that TGF-β signalling is induced later during hindlimb suspension. In fact, the TGF-β signalling pathway has previously been reported to be either unchanged in skeletal muscle after 1–3 days or induced after 7–10 days of unloading [46,68,69]. We also reported an upregulation of *Trim63* and *Fbxo32* during hindlimb suspension. Although these atrogenes are targets of the TGF-β signalling activation, they are also regulated by other signalling pathways [5]. 

We and others reported that the balance between TGF-β and BMP signalling seems crucial for muscle-mass maintenance during catabolic situations [39,44,45,46,70]. Indeed, using the hibernating bear model, we recently reported that TGF-β signalling, i.e., a negative regulator of muscle mass, was downregulated at the transcriptomic level in muscles that are resistant to atrophy, while BMP signalling, i.e., a positive regulator of muscle mass, was maintained [46]. Previous data suggested that TGF-β inhibition would release SMAD4, i.e., the common actor in TGF-β and BMP signalling, which could, thus, be recruited to BMP signalling and promote hypertrophy and/or counteract atrophy [45]. Here, we reported an increase in the SMAD1/5 nuclear protein levels in the halofuginone-treated control mice, suggesting there was concomitant BMP signalling activation and TGF-β inhibition. In addition, BMP activation was reported to increase during denervation, intensive care disuse, and amyotrophic lateral sclerosis and was described as essential to counteract excessive muscle wasting [44,45]. In agreement, we reported here that BMP transcription factors SMAD1/5 accumulated in the nucleus in H_2_O-treated mice but, surprisingly, declined in HF-treated mice after 7 days of hindlimb suspension. Whether the higher basal pools of nuclear SMAD1/5 and their maintenance after 3 days of hindlimb suspension in HF-treated mice contributed to attenuating skeletal muscle atrophy during hindlimb suspension remains to be explored. Of note, BMP signalling has been reported to promote protein synthesis in muscle [44]. Maintenance of the BMP pathway after 3 days of hindlimb suspension may have contributed to the partial preservation of protein synthesis and muscle mass in HF-treated mice. Nuclear translocation of SMAD1/5 represses the transcription of FBXO30/Musa1 [44,45]. However, we did not observe any change in *Fbxo30* expression. Mechanisms by which BMP signalling controls muscle mass are still very poorly understood and will require further studies, particularly with a comprehensive characterisation of the BMP target genes in skeletal muscle. We can also speculate that HF-induced BMP activation has helped to limit muscle atrophy induced by ATF4-regulated atrogenes (Figure 7). 

In conclusion, halofuginone treatment reproduced the muscle features of hibernating bears in gastrocnemius mice muscles with (i) the activation of ATF4-regulated atrogenes and (ii) the concurrent inhibition of TGF-β signalling and promotion of BMP signalling, without resulting in muscle atrophy (Figure 7). These characteristics were associated with mitigated muscle atrophy during physical inactivity. To date, clinical trials have all attempted to inhibit the TGF-β pathway, mostly with side effects or minimal efficiency [71]. Our study suggests halofuginone, as a well-tolerated chemical compound, already used in human clinical trials [36], was able to tune the TGF-β/BMP balance in vivo and likely sustained muscle mass. Moreover, our data open new ways to further decipher by which precise mechanisms ATF4 induces atrophy and how BMP activation can interfere. 

## 4. Materials and Methods

**Ethics, animals housing, and experimental design.** All experiments were conducted with the approval of the regional ethics committee (agreement no. D6334515) following the European Directive 2010/63/EU on the protection of vertebrate animals used for experimental and scientific purposes. This study was performed with 12-week-old C57BL6/J male mice (25–30 g), purchased from Janvier Labs (Le Genest-Saint-Isle, France). They were housed individually upon arrival for 10 days of acclimatisation in a controlled room (22 ± 2 °C, 60 ± 5% humidity, 12 h light/dark cycle, and light period starting at 8 h), fed ad libitum a standard rodent diet (pellets A03 from Safe, Augy, France), and given free access to water. Two distinct animal experiments were performed. To evaluate the effects of a periodic halofuginone (HF) (#32481, Sigma, Saint-Quentin-Fallavier, France) administration, we performed a first protocol where mice received either HF (0.25 µg/g) or water (H_2_O) by gavage 3 times a week for 1 to 4 weeks (n = 6 animals per group). This dose was reported as well tolerated over longer periods [31,33,60]. Gastrocnemius muscle was sampled 6 h after the last HF/H_2_O administration at the end of each week. Subsequently, we performed a second protocol to test whether HF administration before hindlimb unloading had a positive effect on muscle mass and function. For that purpose, we performed two separate animal experiments. In each experiment, mice received either HF (0.25 µg/g) or H_2_O by gavage 3 times a week for 3 weeks and were afterwards subjected either to hindlimb unloading through tail suspension (HS) or kept unsuspended (Ctrl) for 3 or 7 days, as previously described [46] (n = 8–19 animals per group). We did not record any difference between Ctrl mice at 3 or 7 days for all the measurements reported in this manuscript. We, therefore, pooled the two groups of Ctrl mice for further analysis and data representation. Food intake and body weight were recorded throughout the different protocols. Unloading in control mice resulted in only a small body weight loss (<10%) that occurred within the first 3 days concomitantly with a decrease in food intake, whereas HF treatment did not modify food intake or body weight ( see Supplementary Figures S1 and S2).

**Tissue collection.** At the end of the experiments, mice were euthanised by cervical dislocation. The soleus, gastrocnemius, tibialis anterior, and extensor digitorum longus (EDL) muscles were carefully collected and weighed prior to immediate freezing in liquid nitrogen and storage at −80 °C until analyses.

**Measurement of protein synthesis in gastrocnemius.** At the end of protocol 2, mice received an intraperitoneal injection of 0.040 μmol/g puromycin (#P8833, Sigma, Saint-Quentin-Fallavier, France) dissolved in 100 μL of a saline solution before euthanasia, as described previously [72]. At exactly 30 min post-puromycin injection, gastrocnemius muscle was dissected and frozen in liquid nitrogen for Western blot analysis, as follows.

**Histology and morphometric measurements.** A part of the gastrocnemius muscle was collected at the end of protocol 2 and frozen in isopentane chilled with liquid nitrogen and stored at −80 °C until use. Serial muscle cross-sections (10 µm thick) were obtained using a cryostat (HM500M Microm International, Fisher Scientific, Illkirch, France) at −20 °C. Cross-sections were labelled with anti-laminin-α1 (L9393 Sigma, Saint-Quentin-Fallavier, France) to outline the fibre cross-sectional area (CSA) and BFF3 antibody (#AB_2266724, DSHB, Iowa City, IA, USA) to determine myosin heavy chain type 2B fibre. Both were subsequently hybridised with a corresponding secondary antibody conjugated to Alexa-Fluor (Invitrogen, Cergy-Pontoise, France). Image acquisitions were performed with a high-resolution ORCA-Flash4.0 LT+ Digital CMOS camera coupled to a IX-73 microscope (Olympus, Münster, Germany) and Cell-Sens dimension software (Olympus Soft Imaging Solutions, Münster, Germany). The CSA was determined for 1000–1500 fibres per animal, using ImageJ software 1.53f51 (http://rsb.info.nih.gov/ij/, accessed on 3 April 2018).

**Protein isolation.** Gastrocnemius muscles were pulverised in liquid nitrogen. (1) For all targets, ~30 mg of the resulting powders were homogenised using a polytron in 1 mL of an ice-cold buffer (10 mM Tris pH 7.5, 150 mM NaCl, 1 mM EDTA, 1 mM EGTA, 1% Triton X-100, and 0.5% Igepal CA630) containing inhibitors of proteases (Protease Inhibitor Cocktail) and phosphatases (1 mM Na3VO3 and 10 mM NaF) (Sigma, Saint-Quentin-Fallavier, France). The homogenates were stirred for 1h at 4 °C and then centrifuged at 10,000× *g* for 15 min at 4 °C. The resulting supernatants containing total soluble proteins were then stored at −80 °C until use. (2) For SMADs protein level analysis, subcellular fractionation was performed. For that purpose, ~50 mg of gastrocnemius powder samples were homogenised for 1 min on ice using a polytron in 500 μL of ice-cold extraction buffer (10 mM HEPES, pH 7.5, 10 mM MgCl2, 5 mM KCl, 0.1 mM EDTA, pH 8.0, and 0.1% Triton X-100) [73]. The resulting homogenates were subjected to sequential fractionation steps to separate soluble cytosolic and nuclear proteins as described [74]. Pellets containing nuclear proteins were solubilised in nuclear extraction buffer (20 mM HEPES, pH 7.9, 25% glycerol, 500 mM NaCl, 1.5 mM MgCl2, 0.2 mM EDTA, and pH 8.0) [73]. For all protein extracts, protein concentration was determined using the Bradford Protein Assay Kit (Biorad, Marnes-la-Coquette, France). Proteins were then diluted in Laemmli buffer and stored at −80 °C until use.

**Western blots.** Protein contents for (i) SMAD family members (anti-SMAD1-5, PA5-80036, Thermofisher, Illkirch, France; anti-SMAD2-3, #8685, Cell Signalling Technology, Saint-Cyr-L’Ecole, France; anti-SMAD4, ab230815, Abcam, Cambridge, UK), (ii) total and phosphorylated eukaryotic initiation factor 2 alpha (anti-eIF2α, #9722, Cell Signalling Technology; anti-p-Ser51eIF2α, ab32157, and Abcam), and (iii) incorporation of puromycin (anti-puromycin clone 12D10, MABE343, Millipore, Burlington, MA, USA) were assessed by immunoblotting. Briefly, 20–40 µg of protein extracts were subjected to SDS-PAGE (sodium dodecyl sulfate-polyacrylamide gel electrophoresis) using TGX™ FastCast™ 10% Acrylamide gels (Biorad, Marnes-la-Coquette, France) and transferred onto a PVDF membrane (Hybond P, Amersham, England) using Trans-Blot^®^ Turbo™ Transfer System standard protocol (Biorad, Marnes-la-Coquette, France). Western blots were blocked for 1 h at room temperature in TBS (Tris-Buffered Saline) buffer with 0.1% Tween-20 (TBS-T, pH = 7.8) with 5% bovine serum albumin (BSA) for all the targets, in accordance with the instructions of the manufacturer. They were then washed thrice in TBS-T and incubated (overnight, stirring, 4 °C) with appropriate primary antibodies diluted at 1:1000, except for anti-puromycin diluted at 1:5000. Western blots were then washed and incubated for 1 h with an appropriate secondary antibody (HRP-conjugated anti-rabbit (#7074) or anti-mouse (#7076) IgGs) (Cell Signalling Technology, Saint-Cyr-L’Ecole, France). For anti-puromycin antibody, an anti-mouse IgG2Ak (115-035-206, Jackson ImmunoResearch Laboratories, West Grove, PA, USA) was used. Signals were detected after incubation with Luminata Crescendo Western HRP substrate (Millipore, Burlington, MA, USA) and visualised using G: BOX ChemiXT4 (XL1) imaging system (Syngene, Frederick, MD, USA). Signals were then quantified using ImageJ 1.53f51 software. Two samples from each group were loaded on each gel. The signal recorded within each lane of one Western blot was normalised to the overall signal of that blot, and then signals were normalised to the total amount of proteins determined by the Biorad’s stain-free system or ponceau S to correct for uneven loading. The normalised values were then averaged by group and expressed as the fold change from the mean of all H_2_O-ctrl samples.

**RT-qPCR.** Total RNA from gastrocnemius muscle samples was extracted with Macherey-Nagel™ NucleoSpin™ 96 RNA Kit and KingFisher™ Duo Prime Purification System, in accordance with the instructions of the manufacturer (Macherey-Nagel, Hoerdt Cedex, France). RNA was quantified by measuring the absorbance at 260 nm on a NanoDrop ND-1000 spectrophotometer (Thermo Scientific, Wilmington, DE, USA). Then, 500 ng of RNA were treated with DNase I (Invitrogen, Cergy-Pontoise, France) prior to reverse transcription using random primers and SuperScript II (Invitrogen, Cergy-Pontoise, France), in accordance with the instructions of the manufacturer. Real-time PCR was carried out using the CFX96 Real-Time PCR detection system (Biorad, Marnes-la-Coquette, France). Primer sequences are provided in Supplementary Table S1. PCR reactions were performed using the IQ SYBR Green Supermix (Biorad, Marnes-la-Coquette, France), in accordance with the instructions of the manufacturer. The comparative threshold cycle (2ΔΔCT) method was used to compare the relative mRNA expression between each group, using TBP (TATA binding protein) as a reference gene for muscle. The relative mRNA abundance was arbitrarily set to 1 for the H_2_O-Ctrl group.

**Statistics.** All data are means ± SEM and were analysed for normality of residuals using the Shapiro-Wilk test. No set of data was transformed for non-normality distribution. For protocol 1 (n = 6/group), we performed a multiple Welch t-test within each week. For protocol 2 (n = 8–19/group), we performed a two-way ANOVA with the factors “Hindlimb suspension” and “Halofuginone” and corrected the data for multiple comparisons using Tukey’s test. These analyses were performed using Prism 9 (GraphPad Prism 9, San Diego, CA, USA).

**Transcriptomic Data.** We used transcriptomic data from already published studies [46]. The transcriptomic bear data supporting Figure 6 of this study are openly available in the GEO repository database (https://www.ncbi.nlm.nih.gov/geo/query/acc.cgi, reference no. GSE144856, accessed on 1 September 2021). To identify the differentially expressed genes (DEGs) from this list, we selected a winter/summer (FC) > 1.0 with an adjusted *p*-value < 0.05 as cut-off for the up-regulated genes.

## Data Availability

The analysed transcriptomic bear data that support the findings of Figure 6 are openly available in the GEO repository database at https://www.ncbi.nlm.nih.gov/geo/query/acc.cgi, accessed on 16 June 2021, reference no. GSE144856. Other data that support the findings of this study are available in the supplementary materials of this article.

## Supplementary Materials

The supporting information can be downloaded at: https://www.mdpi.com/article/10.3390/ijms24010621/s1.

## References

[1] Alkner B.A., Tesch P.A. (2004). Knee Extensor and Plantar Flexor Muscle Size and Function Following 90 Days of Bed Rest with or without Resistance Exercise. Eur. J. Appl. Physiol..

[2] Cui Q., Yang H., Gu Y., Zong C., Chen X., Lin Y., Sun H., Shen Y., Zhu J. (2020). RNA Sequencing (RNA-Seq) Analysis of Gene Expression Provides New Insights into Hindlimb Unloading-Induced Skeletal Muscle Atrophy. Ann. Transl. Med..

[3] Oliveira J.R.S., Mohamed J.S., Myers M.J., Brooks M.J., Alway S.E. (2019). Effects of Hindlimb Suspension and Reloading on Gastrocnemius and Soleus Muscle Mass and Function in Geriatric Mice. Exp. Gerontol..

[4] Thyfault J.P., Du M., Kraus W.E., Levine J.A., Booth F.W. (2015). Physiology of Sedentary Behavior and Its Relationship to Health Outcomes. Med. Sci. Sport. Exerc..

[5] Peris-Moreno D., Cussonneau L., Combaret L., Polge C., Taillandier D. (2021). Ubiquitin Ligases at the Heart of Skeletal Muscle Atrophy Control. Molecules.

[6] Vainshtein A., Sandri M. (2020). Signaling Pathways That Control Muscle Mass. Int. J. Mol. Sci..

[7] Argilés J.M., Campos N., Lopez-Pedrosa J.M., Rueda R., Rodriguez-Mañas L. (2016). Skeletal Muscle Regulates Metabolism via Interorgan Crosstalk: Roles in Health and Disease. J. Am. Med. Dir. Assoc..

[8] Bonaldo P., Sandri M. (2013). Cellular and Molecular Mechanisms of Muscle Atrophy. Dis. Model. Mech..

[9] Lecker S.H., Goldberg A.L., Mitch W.E. (2006). Protein Degradation by the Ubiquitin–Proteasome Pathway in Normal and Disease States. JASN.

[10] Sacheck J.M., Hyatt J.K., Raffaello A., Thomas Jagoe R., Roy R.R., Reggie Edgerton V., Lecker S.H., Goldberg A.L. (2007). Rapid Disuse and Denervation Atrophy Involve Transcriptional Changes Similar to Those of Muscle Wasting during Systemic Diseases. FASEB J..

[11] Ebert S.M., Monteys A.M., Fox D.K., Bongers K.S., Shields B.E., Malmberg S.E., Davidson B.L., Suneja M., Adams C.M. (2010). The Transcription Factor ATF4 Promotes Skeletal Myofiber Atrophy during Fasting. Mol. Endocrinol..

[12] Ebert S.M., Dyle M.C., Kunkel S.D., Bullard S.A., Bongers K.S., Fox D.K., Dierdorff J.M., Foster E.D., Adams C.M. (2012). Stress-Induced Skeletal Muscle Gadd45a Expression Reprograms Myonuclei and Causes Muscle Atrophy. J. Biol. Chem..

[13] Ebert S.M., Dyle M.C., Bullard S.A., Dierdorff J.M., Murry D.J., Fox D.K., Bongers K.S., Lira V.A., Meyerholz D.K., Talley J.J. (2015). Identification and Small Molecule Inhibition of an Activating Transcription Factor 4 (ATF4)-Dependent Pathway to Age-Related Skeletal Muscle Weakness and Atrophy. J. Biol. Chem..

[14] Taillandier D., Polge C. (2019). Skeletal Muscle Atrogenes: From Rodent Models to Human Pathologies. Biochimie.

[15] Pakos-Zebrucka K., Koryga I., Mnich K., Ljujic M., Samali A., Gorman A.M. (2016). The Integrated Stress Response. EMBO Rep..

[16] Lu Y.-N., Kavianpour S., Zhang T., Zhang X., Nguyen D., Thombre R., He L., Wang J. (2021). MARK2 Phosphorylates EIF2α in Response to Proteotoxic Stress. PLoS Biol..

[17] Fox D.K., Ebert S.M., Bongers K.S., Dyle M.C., Bullard S.A., Dierdorff J.M., Kunkel S.D., Adams C.M. (2014). P53 and ATF4 Mediate Distinct and Additive Pathways to Skeletal Muscle Atrophy during Limb Immobilization. Am. J. Physiol.-Endocrinol. Metab..

[18] Ebert S.M., Bullard S.A., Basisty N., Marcotte G.R., Skopec Z.P., Dierdorff J.M., Al-Zougbi A., Tomcheck K.C., DeLau A.D., Rathmacher J.A. (2020). Activating Transcription Factor 4 (ATF4) Promotes Skeletal Muscle Atrophy by Forming a Heterodimer with the Transcriptional Regulator C/EBPβ. J. Biol. Chem..

[19] Choi R.H., McConahay A., Silvestre J.G., Moriscot A.S., Carson J.A., Koh H.-J. (2019). TRB3 Regulates Skeletal Muscle Mass in Food Deprivation–Induced Atrophy. FASEB J..

[20] Shang G., Han L., Wang Z., Liu Y., Yan S., Sai W., Wang D., Li Y., Zhang W., Zhong M. (2020). Sarcopenia Is Attenuated by TRB3 Knockout in Aging Mice via the Alleviation of Atrophy and Fibrosis of Skeletal Muscles. J. Cachexia Sarcopenia Muscle.

[21] B’chir W., Maurin A.-C., Carraro V., Averous J., Jousse C., Muranishi Y., Parry L., Stepien G., Fafournoux P., Bruhat A. (2013). The EIF2α/ATF4 Pathway Is Essential for Stress-Induced Autophagy Gene Expression. Nucleic Acids Res..

[22] Han S., Zhu L., Zhu Y., Meng Y., Li J., Song P., Yousafzai N.A., Feng L., Chen M., Wang Y. (2021). Targeting ATF4-Dependent pro-Survival Autophagy to Synergize Glutaminolysis Inhibition. Theranostics.

[23] Luhr M., Torgersen M.L., Szalai P., Hashim A., Brech A., Staerk J., Engedal N. (2019). The Kinase PERK and the Transcription Factor ATF4 Play Distinct and Essential Roles in Autophagy Resulting from Tunicamycin-Induced ER Stress. J. Biol. Chem..

[24] Rzymski T., Milani M., Pike L., Buffa F., Mellor H.R., Winchester L., Pires I., Hammond E., Ragoussis I., Harris A.L. (2010). Regulation of Autophagy by ATF4 in Response to Severe Hypoxia. Oncogene.

[25] Kim K.H., Jeong Y.T., Oh H., Kim S.H., Cho J.M., Kim Y.-N., Kim S.S., Kim D.H., Hur K.Y., Kim H.K. (2013). Autophagy Deficiency Leads to Protection from Obesity and Insulin Resistance by Inducing Fgf21 as a Mitokine. Nat. Med..

[26] Kasai S., Yamazaki H., Tanji K., Engler M.J., Matsumiya T., Itoh K. (2019). Role of the ISR-ATF4 Pathway and Its Cross Talk with Nrf2 in Mitochondrial Quality Control. J. Clin. Biochem. Nutr..

[27] Peng W., Robertson L., Gallinetti J., Mejia P., Vose S., Charlip A., Chu T., Mitchell J.R. (2012). Surgical Stress Resistance Induced by Single Amino Acid Deprivation Requires *Gcn2* in Mice. Sci. Transl. Med..

[28] Masiero E., Agatea L., Mammucari C., Blaauw B., Loro E., Komatsu M., Metzger D., Reggiani C., Schiaffino S., Sandri M. (2009). Autophagy Is Required to Maintain Muscle Mass. Cell Metab..

[29] Rodney G.G., Pal R., Abo-Zahrah R. (2016). Redox Regulation of Autophagy in Skeletal Muscle. Free. Radic. Biol. Med..

[30] Memme J.M., Slavin M., Moradi N., Hood D.A. (2021). Mitochondrial Bioenergetics and Turnover during Chronic Muscle Disuse. Int. J. Mol. Sci..

[31] Barzilai-Tutsch H., Genin O., Pines M., Halevy O. (2020). Early Pathological Signs in Young Dysf Mice Are Improved by Halofuginone. Neuromuscul. Disord..

[32] Barzilai-Tutsch H., Bodanovsky A., Maimon H., Pines M., Halevy O. (2016). Halofuginone Promotes Satellite Cell Activation and Survival in Muscular Dystrophies. Biochim. Biophys. Acta (BBA) Mol. Basis Dis..

[33] Barzilai-Tutsch H., Dewulf M., Lamaze C., Butler Browne G., Pines M., Halevy O. (2018). A Promotive Effect for Halofuginone on Membrane Repair and Synaptotagmin-7 Levels in Muscle Cells of Dysferlin-Null Mice. Hum. Mol. Genet..

[34] Mordechay S., Smullen S., Evans P., Genin O., Pines M., Halevy O. (2021). Differential Effects of Halofuginone Enantiomers on Muscle Fibrosis and Histopathology in Duchenne Muscular Dystrophy. Int. J. Mol. Sci..

[35] Roffe S., Hagai Y., Pines M., Halevy O. (2010). Halofuginone Inhibits Smad3 Phosphorylation via the PI3K/Akt and MAPK/ERK Pathways in Muscle Cells: Effect on Myotube Fusion. Exp. Cell Res..

[36] Pines M., Spector I. (2015). Halofuginone—The Multifaceted Molecule. Molecules.

[37] Gnainsky Y., Kushnirsky Z., Bilu G., Hagai Y., Genina O., Volpin H., Bruck R., Spira G., Nagler A., Kawada N. (2007). Gene Expression during Chemically Induced Liver Fibrosis: Effect of Halofuginone on TGF-β Signaling. Cell Tissue Res..

[38] Lokireddy S., Mouly V., Butler-Browne G., Gluckman P.D., Sharma M., Kambadur R., McFarlane C. (2011). Myostatin Promotes the Wasting of Human Myoblast Cultures through Promoting Ubiquitin-Proteasome Pathway-Mediated Loss of Sarcomeric Proteins. Am. J. Physiol. Cell Physiol..

[39] Sartori R., Gregorevic P., Sandri M. (2014). TGFβ and BMP Signaling in Skeletal Muscle: Potential Significance for Muscle-Related Disease. Trends Endocrinol. Metab..

[40] Sartori R., Milan G., Patron M., Mammucari C., Blaauw B., Abraham R., Sandri M. (2009). Smad2 and 3 Transcription Factors Control Muscle Mass in Adulthood. Am. J. Physiol. Cell Physiol..

[41] Lee S.-J., Reed L.A., Davies M.V., Girgenrath S., Goad M.E.P., Tomkinson K.N., Wright J.F., Barker C., Ehrmantraut G., Holmstrom J. (2005). Regulation of Muscle Growth by Multiple Ligands Signaling through Activin Type II Receptors. Proc. Natl. Acad. Sci. USA.

[42] Chen J.L., Walton K.L., Hagg A., Colgan T.D., Johnson K., Qian H., Gregorevic P., Harrison C.A. (2017). Specific Targeting of TGF-β Family Ligands Demonstrates Distinct Roles in the Regulation of Muscle Mass in Health and Disease. Proc. Natl. Acad. Sci. USA.

[43] Weiss A., Attisano L. (2013). The TGFbeta Superfamily Signaling Pathway. Wiley Interdiscip. Rev. Dev. Biol..

[44] Winbanks C.E., Chen J.L., Qian H., Liu Y., Bernardo B.C., Beyer C., Watt K.I., Thomson R.E., Connor T., Turner B.J. (2013). The Bone Morphogenetic Protein Axis Is a Positive Regulator of Skeletal Muscle Mass. J. Cell Biol..

[45] Sartori R., Schirwis E., Blaauw B., Bortolanza S., Zhao J., Enzo E., Stantzou A., Mouisel E., Toniolo L., Ferry A. (2013). BMP Signaling Controls Muscle Mass. Nat. Genet..

[46] Cussonneau L., Boyer C., Brun C., Deval C., Loizon E., Meugnier E., Gueret E., Dubois E., Taillandier D., Polge C. (2021). Concurrent BMP Signaling Maintenance and TGF-β Signaling Inhibition Is a Hallmark of Natural Resistance to Muscle Atrophy in the Hibernating Bear. Cells.

[47] Keller T.L., Zocco D., Sundrud M.S., Hendrick M., Edenius M., Yum J., Kim Y.-J., Lee H.-K., Cortese J.F., Wirth D.F. (2012). Halofuginone and Other Febrifugine Derivatives Inhibit Prolyl-TRNA Synthetase. Nat. Chem. Biol..

[48] Luo Y., Xie X., Luo D., Wang Y., Gao Y. (2017). The Role of Halofuginone in Fibrosis: More to Be Explored?. J. Leukoc. Biol..

[49] Hill C.S. (2016). Transcriptional Control by the SMADs. Cold Spring Harb. Perspect. Biol..

[50] Trendelenburg A.U., Meyer A., Rohner D., Boyle J., Hatakeyama S., Glass D.J. (2009). Myostatin Reduces Akt/TORC1/P70S6K Signaling, Inhibiting Myoblast Differentiation and Myotube Size. Am. J. Physiol. Cell Physiol..

[51] Winbanks C.E., Weeks K.L., Thomson R.E., Sepulveda P.V., Beyer C., Qian H., Chen J.L., Allen J.M., Lancaster G.I., Febbraio M.A. (2012). Follistatin-Mediated Skeletal Muscle Hypertrophy Is Regulated by Smad3 and MTOR Independently of Myostatin. J. Cell Biol..

[52] Tinker D.B., Harlow H.J., Beck T.D.I. (1998). Protein Use and Muscle-Fiber Changes in Free-Ranging, Hibernating Black Bears. Physiol. Zool..

[53] Hershey J.D., Robbins C.T., Nelson O.L., Lin D.C. (2008). Minimal Seasonal Alterations in the Skeletal Muscle of Captive Brown Bears. Physiol. Biochem. Zool..

[54] Lohuis T.D., Harlow H.J., Beck T.D.I. (2007). Hibernating Black Bears (Ursus Americanus) Experience Skeletal Muscle Protein Balance during Winter Anorexia. Comp. Biochem. Physiol. Part B Biochem. Mol. Biol..

[55] Chen D., Wang Y., Chin E.R. (2015). Activation of the Endoplasmic Reticulum Stress Response in Skeletal Muscle of G93A*SOD1 Amyotrophic Lateral Sclerosis Mice. Front. Cell. Neurosci..

[56] Bohnert K.R., Gallot Y.S., Sato S., Xiong G., Hindi S.M., Kumar A. (2016). Inhibition of ER Stress and Unfolding Protein Response Pathways Causes Skeletal Muscle Wasting during Cancer Cachexia. FASEB J..

[57] Ebert S.M., Rasmussen B.B., Judge A.R., Judge S.M., Larsson L., Wek R.C., Anthony T.G., Marcotte G.R., Miller M.J., Yorek M.A. (2022). Biology of Activating Transcription Factor 4 (ATF4) and Its Role in Skeletal Muscle Atrophy. J. Nutr..

[58] Turgeman T., Hagai Y., Huebner K., Jassal D.S., Anderson J.E., Genin O., Nagler A., Halevy O., Pines M. (2008). Prevention of Muscle Fibrosis and Improvement in Muscle Performance in the Mdx Mouse by Halofuginone. Neuromuscul. Disord..

[59] Bodanovsky A., Guttman N., Barzilai-Tutsch H., Genin O., Levy O., Pines M., Halevy O. (2014). Halofuginone Improves Muscle-Cell Survival in Muscular Dystrophies. Biochim. Biophys. Acta (BBA) Mol. Cell Res..

[60] Murphy A.P., Greally E., O’Hogain D., Blamire A., Caravan P., Straub V. (2021). Use of EP3533-Enhanced Magnetic Resonance Imaging as a Measure of Disease Progression in Skeletal Muscle of Mdx Mice. Front. Neurol..

[61] Bongers K.S., Fox D.K., Ebert S.M., Kunkel S.D., Dyle M.C., Bullard S.A., Dierdorff J.M., Adams C.M. (2013). Skeletal Muscle Denervation Causes Skeletal Muscle Atrophy through a Pathway That Involves Both Gadd45a and HDAC4. Am. J. Physiol. Endocrinol. Metab..

[62] Harding H.P., Zhang Y., Zeng H., Novoa I., Lu P.D., Calfon M., Sadri N., Yun C., Popko B., Paules R. (2003). An Integrated Stress Response Regulates Amino Acid Metabolism and Resistance to Oxidative Stress. Mol. Cell.

[63] Iurlaro R., Püschel F., León-Annicchiarico C.L., O’Connor H., Martin S.J., Palou-Gramón D., Lucendo E., Muñoz-Pinedo C. (2017). Glucose Deprivation Induces ATF4-Mediated Apoptosis through TRAIL Death Receptors. Mol. Cell. Biol..

[64] Qing G., Li B., Vu A., Skuli N., Walton Z.E., Liu X., Mayes P.A., Wise D.R., Thompson C.B., Maris J.M. (2012). ATF4 Regulates MYC-Mediated Neuroblastoma Cell Death upon Glutamine Deprivation. Cancer Cell.

[65] B’chir W., Chaveroux C., Carraro V., Averous J., Maurin A.-C., Jousse C., Muranishi Y., Parry L., Fafournoux P., Bruhat A. (2014). Dual Role for CHOP in the Crosstalk between Autophagy and Apoptosis to Determine Cell Fate in Response to Amino Acid Deprivation. Cell. Signal..

[66] Lin J.H., Li H., Zhang Y., Ron D., Walter P. (2009). Divergent Effects of PERK and IRE1 Signaling on Cell Viability. PLoS ONE.

[67] Duan M., Wei X., Cheng Z., Liu D., Fotina H., Xia X., Hu J. (2020). Involvement of EIF2α in Halofuginone-Driven Inhibition of TGF-Β1-Induced EMT. J. Biosci..

[68] Yoshihara T., Takaragawa M., Dobashi S., Naito H. (2022). Losartan Treatment Attenuates Hindlimb Unloading-Induced Atrophy in the Soleus Muscle of Female Rats via Canonical TGF-β Signaling. J. Physiol. Sci..

[69] Hirose T., Nakazato K., Song H., Ishii N. (2008). TGF-β _1_ and TNF-α Are Involved in the Transcription of Type I Collagen α _2_ Gene in Soleus Muscle Atrophied by Mechanical Unloading. J. Appl. Physiol..

[70] Sartori R., Hagg A., Zampieri S., Armani A., Winbanks C.E., Viana L.R., Haidar M., Watt K.I., Qian H., Pezzini C. (2021). Perturbed BMP Signaling and Denervation Promote Muscle Wasting in Cancer Cachexia. Sci. Transl. Med..

[71] Suh J., Lee Y.-S. (2020). Myostatin Inhibitors: Panacea or Predicament for Musculoskeletal Disorders?. J. Bone Metab..

[72] Goodman C.A., Hornberger T.A. (2013). Measuring Protein Synthesis With SUnSET: A Valid Alternative to Traditional Techniques?. Exerc. Sport Sci. Rev..

[73] Blough E., Dineen B., Esser K. (1999). Extraction of Nuclear Proteins from Striated Muscle Tissue. BioTechniques.

[74] Dimauro I., Pearson T., Caporossi D., Jackson M.J. (2012). A Simple Protocol for the Subcellular Fractionation of Skeletal Muscle Cells and Tissue. BMC Res. Notes.

