# Supplementary material for: Induction of ATF4-Regulated Atrogenes Is Uncoupled from Muscle Atrophy during Disuse in Halofuginone-Treated Mice and in Hibernating Brown Bears
Source: Int J Mol Sci. 2022 Dec 30;24(1):621. doi: 10.3390/ijms24010621 (PMC9820832; doi:10.3390/ijms24010621)
Supplement: Supplementary file 1 [file ijms-24-00621-s001.zip › Supplemantary Figures.pdf]

**Supplementary Figure S1**

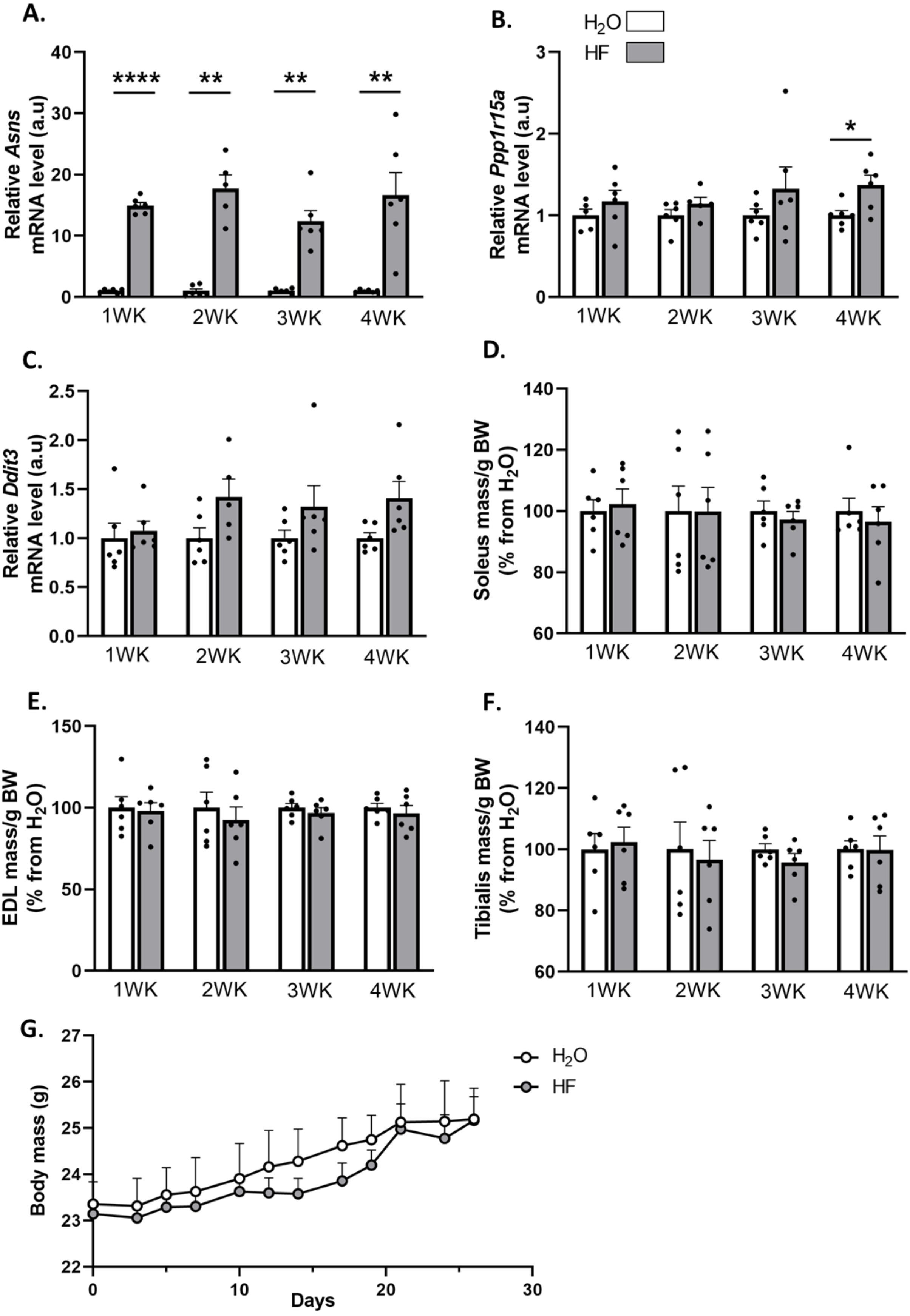

**Supplementary Figure S1. Effect of halofuginone treatment on muscle mass.** Mice were treated with $H_2O$ (white bars) or HF (0.25 µg/g, grey bars) 3 times a week up to 4 weeks (WK) as described in Figure 1A. Muscles were collected 6h after the last HF administration at the end of each week. (A-C) Relative mRNA levels in gastrocnemius for *Asns*, *Ppp1r15a* and *Ddit3* were measured by RT-qPCR. Data were normalized using *Tbp*. Data are expressed as fold change vs. $H_2O$ within each week and presented as individual values with mean bars ± SEM. (D-F) Soleus, Tibialis anterior and Extensor digitorum longus (EDL) mass per gram of body weight (BW). Data are expressed as a percentage from $H_2O$0 within each week and presented as individual values with mean bars ± SEM. (G) Body mass in grams (g) of $H_2O$ (white circles) or HF (grey circles) treated mice. Data are presented as means ± SEM. Statistics are described in Methods. * $p_{adj} < 0.05$; ** $p_{adj} < 0.01$; **** $p_{adj} < 0.0001$.

# Supplementary Figure S2

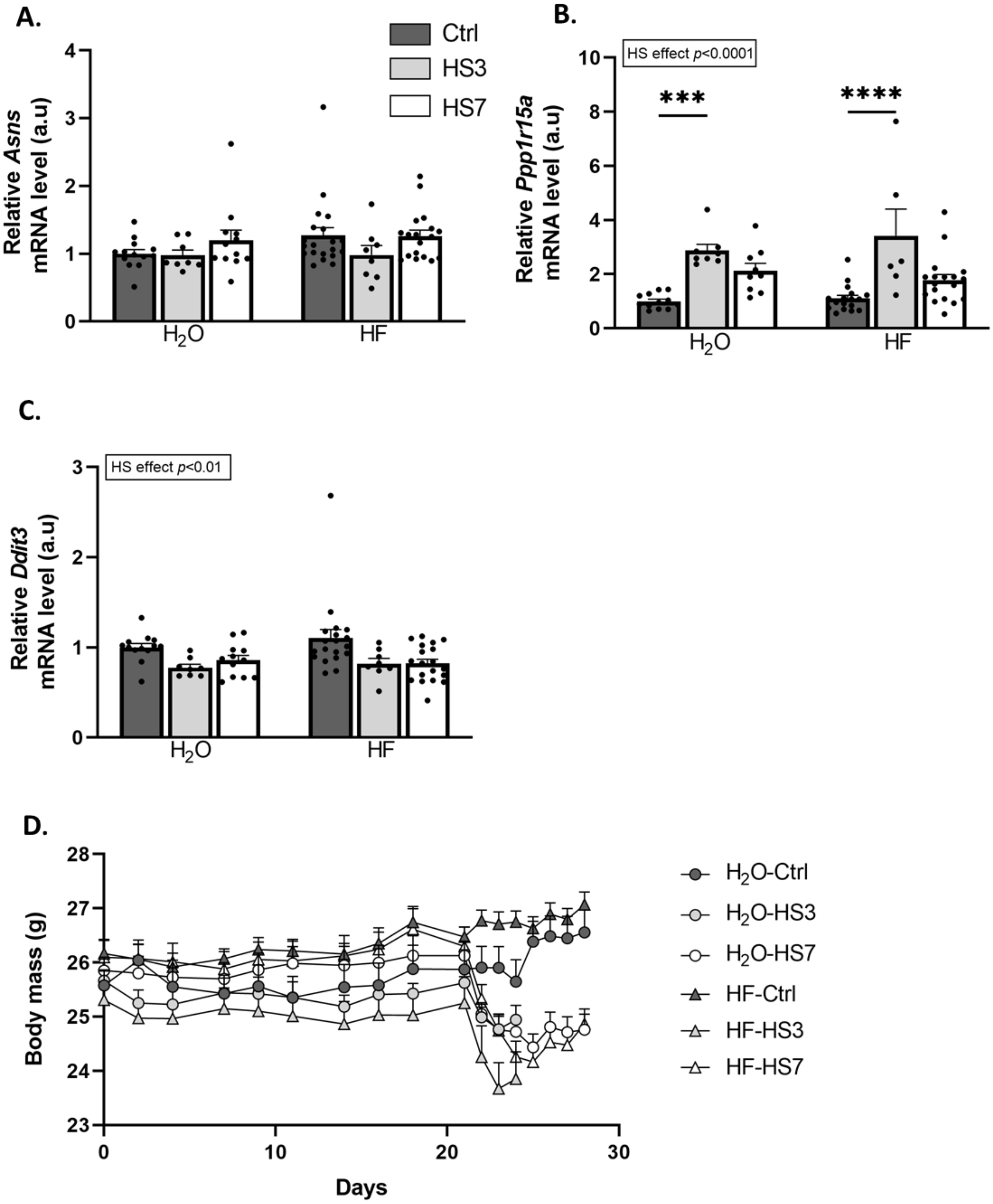

**Supplementary Figure S2. ATF4-regulated alternative target genes expression in muscle during hindlimb suspension.** Mice were treated with $H_2O$ or halofuginone (HF) oral administration (0.25µg/g) 3 times a week for 3 weeks and were then subjected to hindlimb suspension for 3 or 7 days (HS3 and HS7, light grey and white bars, respectively) or kept unsuspended (Ctrl, dark grey bars). (A-C) Relative mRNA levels in gastrocnemius for *Asns*, *Ppp1r15a* and *Ddit3* were measured by RT-qPCR. Data were normalized using *Tbp*. Data are expressed as fold change vs. $H_2O$-Ctrl and presented as individual values with mean bars ± SEM. Statistics are described in Methods. *** $p_{adj}$ <0.001; **** $p_{adj}$ <0.0001. (D) Body mass in grams (g) of $H_2O$ (circles) or HF (triangles) treated mice unsuspended (Ctrl, dark grey) or suspended for 3 or 7 days (HS3 and HS7, light grey and white, respectively). Data are presented as mean ± SEM.

**Supplementary Figure S3**

**A.**

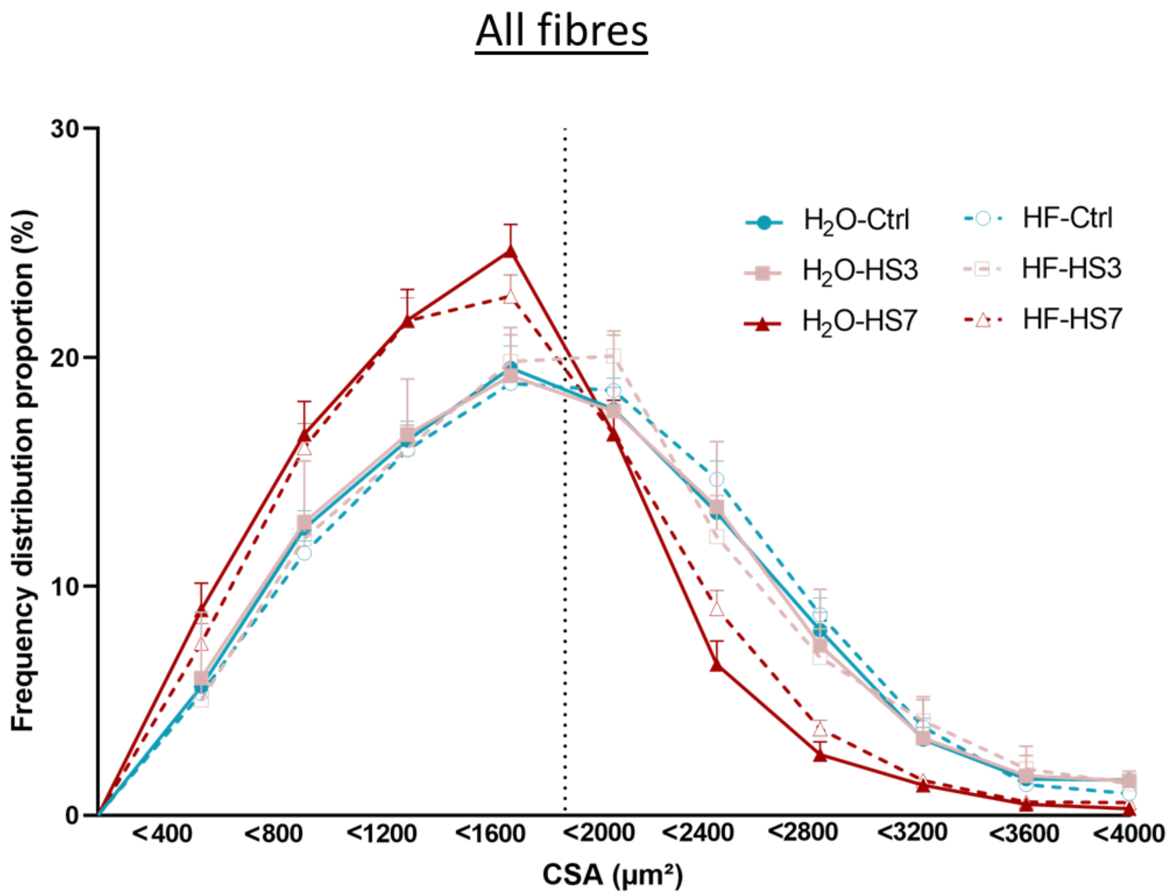


**B.**

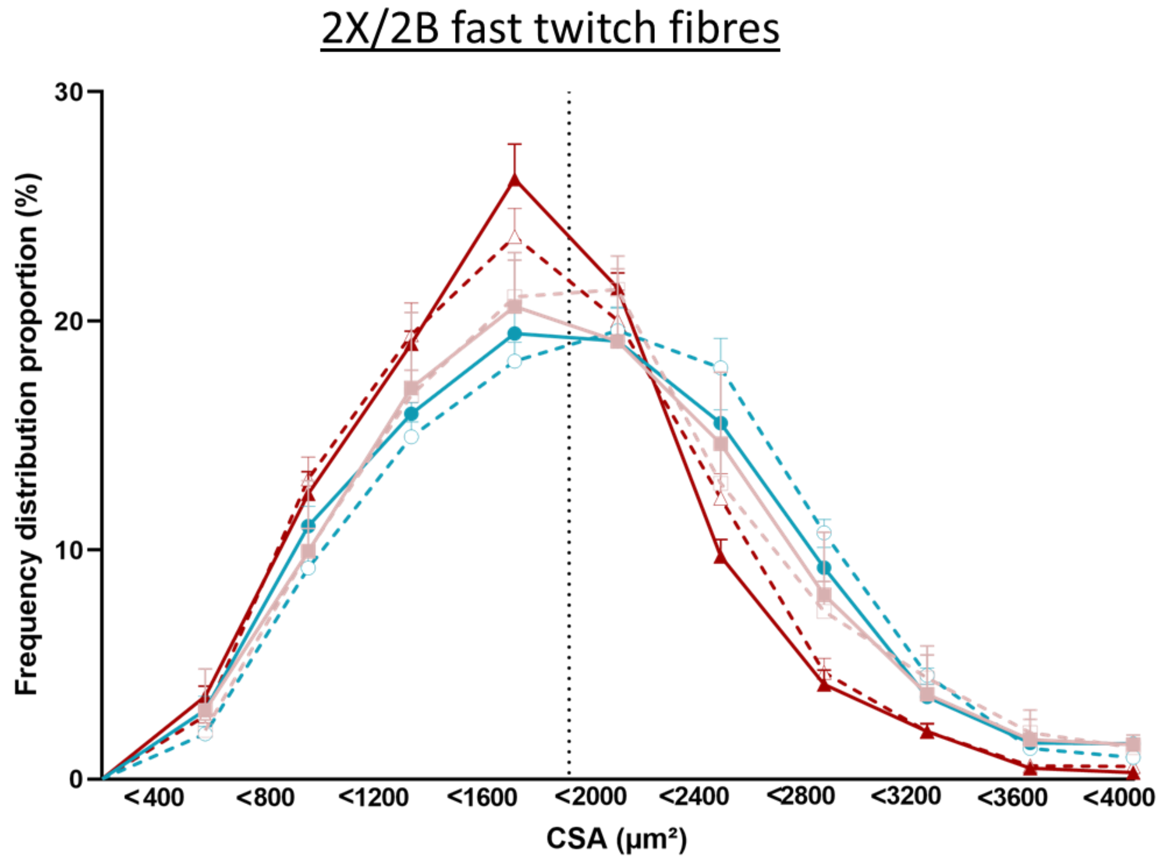

**Supplementary Figure S3. Effects of halofuginone treatment prior to hindlimb suspension on skeletal muscle.** Mice were treated with $H_2O$ or halofuginone (HF, 0.25µg/g) 3 times a week for 3 weeks and were then subjected to hindlimb suspension for 3 or 7 days (HS3 and HS7, respectively) or kept unsuspended (Ctrl) as described in Figure 2A. (A-B) Frequency distribution proportion of fibres cross-sectional area (CSA) in Ctrl (blue circle), HS3 (pink square) or HS7 (red triangle) of mice treated with H2O (filled forms and lines) or HF (empty forms and dotted lines), for all fibres type (A) or 2X/2B fast twitch fibres (B). Data are means ± SEM.

# Supplementary Figure S4

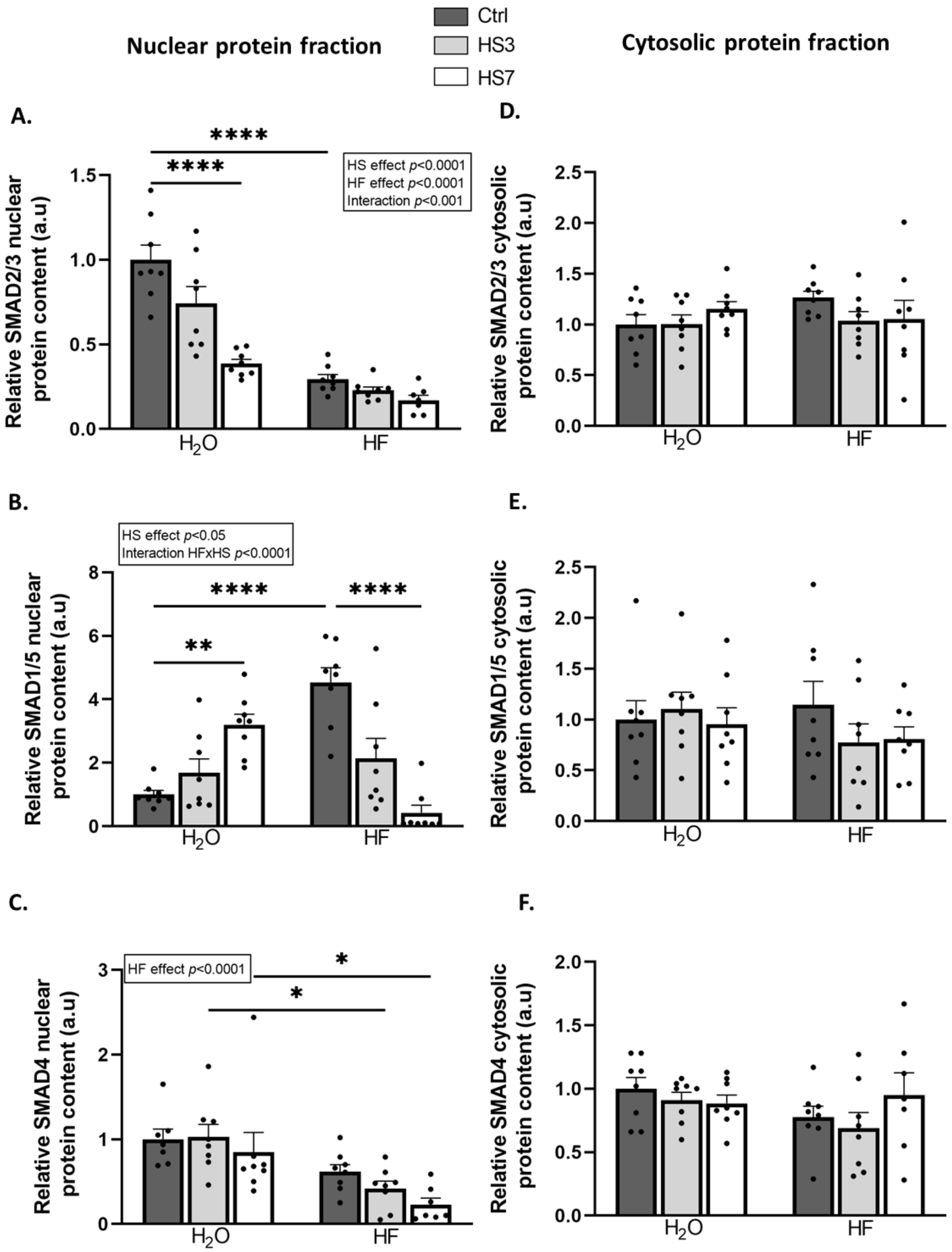

**Supplementary Figure S4**. **Effect of halofuginone treatment prior to hindlimb suspension on SMADs protein content in gastrocnemius muscle.** Mice were treated with $H_2O$ or halofuginone (HF, 0.25µg/g) 3 times a week for 3 weeks and were then subjected for 3 or 7 days (HS3 and HS7, light grey and white bars, respectively) or kept unsuspended (Ctrl, dark grey bars) as described in Figure 2A. (A-F) Relative SMAD2/3, SMAD1/5 and SMAD4 protein levels in gastrocnemius muscle were assessed by Western blotting in the nuclear (A-C) and the cytosolic (D-F) protein fractions;. They were then quantified and normalized to the total protein content. Data are expressed as fold change vs. $H_2O$-Ctrl and presented as individual values with mean bars ± SEM. Statistics are described in Methods. * $p_{adj}$ <0.05; ** $p_{adj}$ <0.01; **** $p_{adj}$ <0.0001.

# Supplementary Figure S5

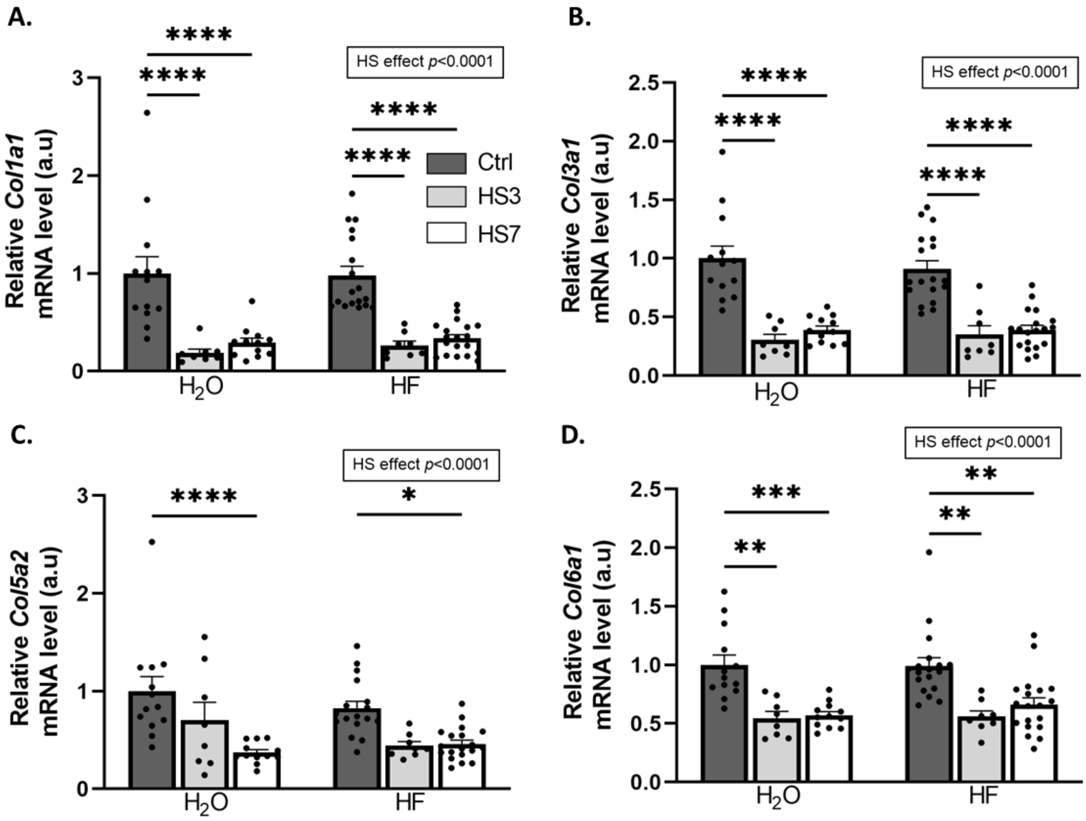


**Supplementary Figure S5**. **Effect of halofuginone treatment prior to hindlimb suspension on collagens expression in gastrocnemius muscle.** Mice were treated with $H_2O$ or halofuginone (HF) oral administration (0.25µg/g) 3 times a week for 3 weeks and were then subjected to hindlimb suspension for 3 or 7 days (HS3 and HS7, light grey and white bars, respectively) or kept unsuspended (Ctrl, dark grey bars). (A-D) Relative mRNA levels in gastrocnemius for *Col1a1*, *Col3a1, Col5a2* and *Col6a1* were measured by RT-qPCR. Data were normalized using *Tbp*. Data are expressed as fold change vs. $H_2O$-Ctrl and presented as individual values with mean bars ± SEM. Statistics are described in Methods. * $p_{adj}$ <0.05; ** $p_{adj}$ <0.01; *** $p_{adj}$ <0.001; **** $p_{adj}$ <0.0001.
